# A novel connexin 50 gene (gap junction protein, alpha 8) mutation associated with congenital nuclear and zonular pulverulent cataract

**Published:** 2013-04-05

**Authors:** Jinyu Li, Qiwei Wang, Qiuyue Fu, Yanan Zhu, Yi Zhai, Yinhui Yu, Kai Zhang, Ke Yao

**Affiliations:** 1 Eye Center of the 2nd Affiliated Hospital, Medical College of Zhejiang University, Hangzhou, China; 2 Zhejiangn provincial key lab of ophthalmology, China; 3 Central hospital of Taizhou, Taizhou, Zhejiang Province, China

## Abstract

**Purpose:**

To characterize the disease-causing mutations in four generations of a Chinese family affected with bilateral congenital nuclear and zonular pulverulent cataract.

**Methods:**

Detailed family history and clinical data were recorded. The phenotype was documented using slit-lamp photography. Candidate genes were amplified using PCR and screened for mutations using bidirectional sequencing.

**Results:**

Affected individuals had nuclear and zonular pulverulent cataract with Y-sutural opacities. Sequencing of the candidate genes revealed a heterozygous c. 139G>C change in the coding sequence of the connexin 50 gene (gap junction protein, alpha 8 [GJA8]), which results in the substitution of a wild-type aspartic acid with a histidine (D47H). This mutation cosegregated with all affected individuals in the family and was not found in unaffected family members or in 100 unrelated controls.

**Conclusions:**

Our study has identified a novel connexin 50 gene (GJA8) mutation, resulting in the amino substitution p. D47H in a Chinese family with nuclear and zonular pulverulent congenital cataracts. This mutation is probably the causative lesion for the observed phenotype in this family.

## Introduction

Congenital cataract, defined as any opacity of the lens present within the first year of life, is responsible for approximately one-tenth of childhood blindness worldwide [1]. Depending on regional socioeconomic development, prevalence varies from one to six cases per 10,000 live births in industrialized countries [2, 3] to five to 15 per 10,000 in the poorest areas of the world [1, 4]. In the poorest areas, the major reason for isolated congenital cataracts is virus infection, including rubella and influenza. In these cases, hygiene standards and vaccination must be improved to prevent congenital cataract [5]. Congenital cataract can occur in an isolated fashion or as one component of a syndrome affecting multiple tissues. Between 8.3% and 25% of congenital cataracts are believed to be inherited [6]. Although autosomal recessive and X-linked forms of inheritance exist, autosomal dominance is the major form of inheritance of congenital cataracts [7].

Up to now, at least 35 independent loci, including more than 20 genes on different chromosomes, have been associated with isolated autosomal dominant congenital cataract (ADCC) [8]. Of the cataract mutations reported to date, about half have mutations in the crystallin genes and a quarter in connexin genes, with the remainder divided among genes that encode heat shock transcription factor-4 (*HSF4*), aquaporin-0 (*AQP0, MIP*), v-maf musculoaponeurotic fibrosarcoma oncogene homolog (*MAF*), paired-like homeodomain 3 (*PITX3*), beaded filament structural protein-2 (*BFSP2*), chromatin modifying protein (*CHMP4B*), lens intrinsic membrane protein 2 (*LIM2*), and other genes [9, 10]. The crystallin and connexin genes appear to be the most commonly associated with congenital cataract. Therefore, it is suitable to consider these genes the top candidates for developing congenital cataract screening strategies. In this study, we investigated a four-generation family with congenital nuclear and zonular pulverulent cataracts and detected a novel missense mutation in the connexin 50 gap junction protein, alpha 8 (*GJA8*) gene that cosegregated with the disease in the family.

## Methods

### Family data and DNA specimens

**Figure 1:**
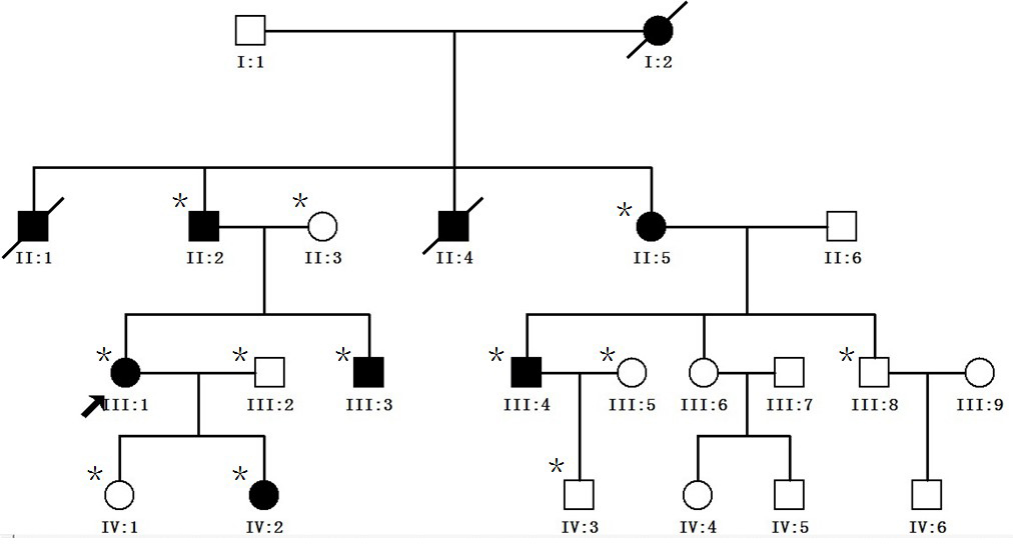
Pedigree of autosomal dominant congenital cataract (ADCC). The proband is marked with an arrow. Squares and circles indicate men and women, respectively. Black and white symbols represent affected and unaffected individuals, respectively. The asterisks indicate family members who were enrolled in this study.

**Figure 2:**
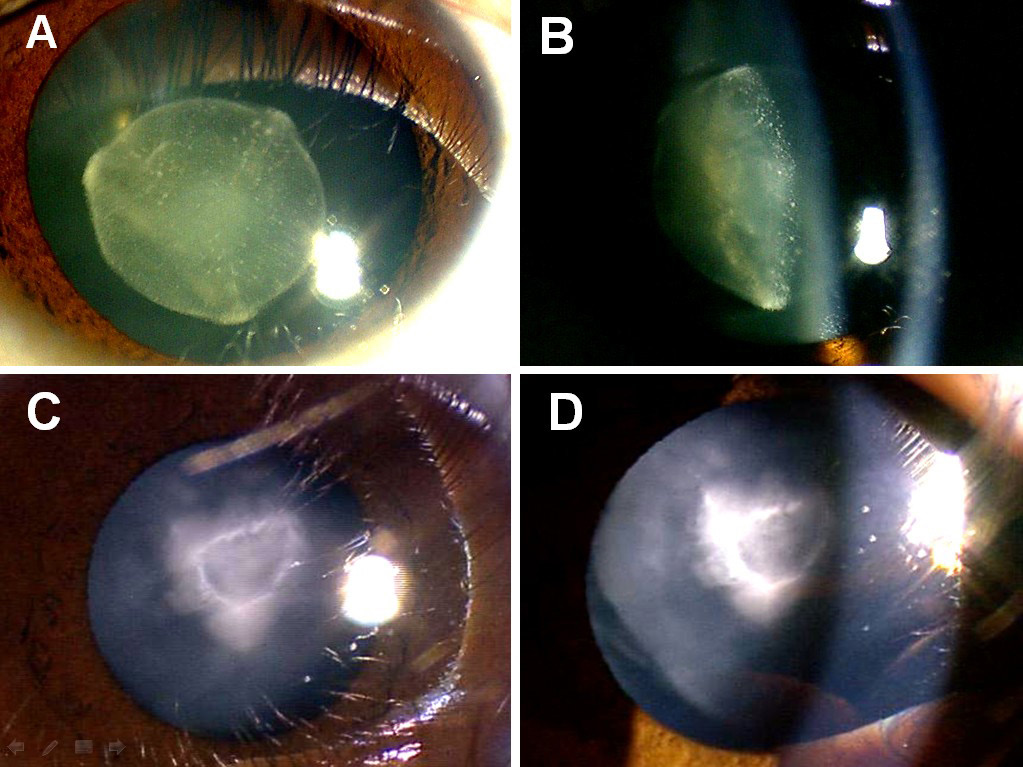
Slit-lamp photograph of the family members who had congenital nuclear and zonular pulverulent cataracts with Y-sutural opacities. The affected member IV:2 (**C**, **D**) had more severe nuclear opacities than her mother III:1 (**A**, **B**).

The four generations of the family from south part of Zhejiang province with ADCC were recruited from the Eye Center of Affiliated Second Hospital, College of Medicine, Zhejiang University, Hangzhou, China in January 2012. In total, 12 individuals, age from six to fifty four years old, participated: six affected and six unaffected, six male and six female. Of the 12, six were spouses (Figure 1). Among the affected individuals,II:2 and II:5 had undergone lens surgery, the other four had bad visual acuity. In addition, 100 munrelated control subjects were recruited. Informed consent in accordance with the Zhejiang Institutional Review Board was obtained from all participants, and the study protocol adhered to the tenets of the Declaration of Helsinki. Detailed medical histories were obtained by interviewing all the 12 individuals in the ADCC family. All participants underwent ophthalmologic examinations, including visual acuity and slit-lamp examination with dilated pupils. Phenotypes were documented with slit-lamp photography (Figure 2). Blood samples were obtained by venipuncture and collected in Becton-Dickinson (BD, Franklin, NJ) Vacutainer tubes containing EDTA. Leukocyte genomic DNA was extracted using the QIAmp Blood kit (Qiagen, Duesseldorf, Germany).

### Mutation screening

We used the functional candidate gene analysis approach. Genomic DNA samples from affected and unaffected members of the family were screened for mutations in *CRYAA*, *CRYAB*, *CRYBA3/1*, *CRYBB2, CRYGC, CRYGD,* connexin 46, and connexin 50 genes using direct sequencing. The coding regions of the candidate genes were amplified with PCR using previously published primer sequences (Table 1) [11-15]. The cycling conditions for PCR were 95 °C preactivation for 5 min, 10 cycles of touchdown PCR with 0.5 °C down per cycle from 60 °C to 55 °C, followed by 30 cycles with denaturation at 95 °C for 25 s, annealing at 55 °C for 25 s, and extension at 72 °C for 40 s. PCR products were isolated by electrophoresis on 3% agarose gels and sequenced using the BigDye Terminator Cycle sequencing kit V 3. 1 (ABI Applied Biosystems; Sangon Co, Shanghai, China) on an ABI PRISM 3730 Sequence Analyzer (ABI), according to the manufacturer’s instructions. Sequencing results were analyzed using Chromas 1.62 and compared with sequences from the NCBI GenBank (*CRYAA*: 21q22.3; NM_000394, *CRYAB*: 11q22; NG_009824;*CRYBA3/1*: 17q11-q12; NM_005208, *CRYBB2*: 22q11. 2; NM_005208, *CRYGC*: 2q33-q35; NM_020989, *CRYGD*: 2q33-q35; NM_006891.3, connexin 46: 13q11-q13; NM_021954, and connexin 50:1q21-q25).

**Table 1:** Polymerase chain reaction primers and product sizes.

Name	Primer sequence (5′-3′)	Product size (bp)	Reference
**Cx50**			[13]
Exon-2–1 F	5′ CAGATATTGACTCAGGGTTG 3′	542	
Exon-2–1R	5′ GATGATGTGGCAGATGTAGG 3′		
Exon-2–2 F	5′ GGCAGCAAAGGCACTAAG 3′	465	
Exon-2–2 R	5′ CTCCACCATCCCAACCTC 3′		
Exon-2–3 F	5′ ATCGTTTCCCACTATTTCC 3′	492	
Exon-2–3 R	5′ GGCGTCACTTCATACGGTTA 3′		
**Cx46**			[13]
Exon-1–1 F	5′ CTCTTCTGGCTCTGGCTTCC 3′	741	
Exon-1–1R	5′ CACCTCGAACAGCGTCTTGA 3′		
Exon-1–2 F	5′ CTTCCCCATCTCCCACATCC 3′	749	
Exon-1–2 R	5′ GGTGGCCGTTGTAGAGCTTG 3′		
Exon-1–3 F	5′ TCCGCCAAGCTCTACAACG 3′	535	
Exon-1–3 R	5′ GAAACCTGATCTCTCCTCCAT 3′		
**CRYBA3/1**			[14]
Exon-1 F	5′GGCAGAGGGAGAGCAGAGTG 3′	207	
Exon-1 R	5′CACTAGGCAGGAGAACTGGG 3′		
Exon-2 F	5′AGTGAGCAGCAGAGCCAGAA 3′	293	
Exon-2 R	5′GGTCAGTCACTGCCTTATGG 3′		
Exon-3 F	5′AAGCACAGAGTCAGACTGAAGT 3′	269	
Exon-3 R	5′CCCCTGTCTGAAGGGACCTG 3′		
Exon-4 F	5′GTACAGCTCTACTGGGATTG 3′	357	
Exon-4 R	5′ACTGATGATAAATAGCATGAACT 3′		
Exon-5 F	5′GAATGATAGCCATAGCACTAG 3′	290	
Exon-5 R	5′TACCGATACGTATGAAATCTGA 3′		
Exon-6 F	5′CATCTCATACCATTGTGTTGAG 3′	295	
Exon-6 R	5′GCAAGGTCTCATGCTTGAGG 3′		
**CRYAA**			[15]
Exon-1 F	5′CTTAATGCCTCCATTCTGCT 3′	593	
Exon-1 R	5′TGGCTGGTGCCTTACAAA 3′		
Exon-2 F	5′ CACCTGACCATAGCCAAACAAC 3′	512	
Exon-2 R	5′ TCTCCCAGGGTTGAAGGCA 3′		
Exon-3 F	5′ GGGGCATGAATCCATAAATC 3′	487	
Exon-3 R	5′ GGAAGCAAAGGAAGACAGACAC 3′		
**CRYAB**			[15]
Exon-1 F	5′ AACCCCTGACATCACCATTC 3′	469	
Exon-1 R	5′ GGAGGAAGGCACTAGCAACC 3′		
Exon-2 F	5′ TGCAGAATAAGACAGCACCTG 3′	296	
Exon-2 R	5′ AATGTAGCCAGCCTCCAAAG 3′		
Exon-3 F	5′ TCTGCCTCTTTCCTCATT 3′	473	
Exon-3 R	5′ CCTTGGAGCCCTCTAAAT 3′		
**CRYBB2**			[12]
Exon-2 F	5′ TGCTCTCTTTCTTTGAGTAGACCTC 3′	385	
Exon-2 R	5′CCCATTTTACAGAAGGGCAAC 3′		
Exon-3 F	5′ ACCCTTCAGCATCCTTTG G 3′	314	
Exon-3 R	5′ GCAGACAGGAGCAAGGGTAG 3′		
Exon-4 F	5′ GCTTGGAGTGGAACTGACCTG 3′	244	
Exon-4 R	5′ GGCAGAGAGAGAAAGTAGGATGATG 3′		
Exon-5 F	5′ GCCCCCTCACCCATACTC 3′	242	
Exon-5 R	5′ CCCCAGAGTCTCAGTTTCCTG 3′		
Exon-6 F	5′ CCTAGTGGCTTATGGATGCTC 3′	347	
Exon-6 R	5′ TCTTCACTTGGAGGTCTGGAG 3′		
**CRYGC**			[11]
Exon-1. 2 F	5′ TGCATAAAATCCCCTTACCGCTGA 3′	522	
Exon-1. 2 R	5′ ACTCTGGCGGCATGATGGAAATC 3′		
Exon-3 F	5′AGACTCATTTGCTTTTTTCCATCCTTCTTTC 3′	407	
Exon-3 R	5′GAAAGAATGACAGAAGTCAGCAATTGCC 3′		
**CRYGD**			[11]
Exon-1. 2 F	5′ CCTCGCCTTGTCCCGC 3′	340	
Exon-1. 2 R	5′ TTAACTTTTGCTTGAAACCATCCA 3′		
Exon-3 F	5′ TGCTTTTCTTCTCTTTTTATTTCTGGGTCC 3′	400	
Exon-3 R	5′AGTAAAGAAAGACACAAGCAAATCAGTGCC 3′		

### Bioinformatics analysis

Computational algorithms are effective in predicting whether a specific amino acid substitution of a protein sequence is deleterious or neutral to the function of the protein. Among various methods, Polymorphism Phenotyping (PolyPhen), Grantham score difference (Align-GVGD), and Sorting Intolerant from Tolerant (SIFT) amino acid substitutions are most commonly used.

PolyPhen takes into account the evolutionary conservation of the amino acid subjected to the mutation and the physicochemical characteristics of the wild-type and mutated amino acid residue [16]. Grantham Variation (GV) measures the degree of biochemical variation among amino acids found at a given position in the multiple sequence alignment. Grantham Deviation (GD) reflects the biochemical distance of the mutant amino acid from the observed amino acid at a particular position (given by GV). Align-GVGD can be used to predict the transactivation activity of each missense substitution [17]. SIFT uses sequence homology to predict whether an amino acid change will affect protein function and potentially contribute to a disease. SIFT, which assigns scores from 0 to 1, predicts substitutions with scores less than 0.05 as deleterious, whereas those greater than or equal to 0.05 are considered tolerated [18].

## Results

### Clinical evaluation

We identified a four-generation Chinese family with a clear diagnosis of ADCC (Figure 1). All affected patients had bilateral nuclear and zonular pulverulent cataracts with Y-sutural opacities, but the degree of lens opacity varied slightly. The affected member IV:2 (Figure 2C,D), a six-year-old girl, had more severe nuclear opacities than her mother, the proband III:1 (Figure 2A,B). The visual acuity of the affected individuals ranged from 0.6 to 0.1; most affected individuals observed visual impairment in their 20s, and then their visual acuity decreased gradually. The affected member IV:2 had the worst visual acuity in childhood, and was diagnosed before the age of three, so she underwent cataract surgery in July 2012. There was no family history of other ocular or systemic abnormalities.

### Mutation screening

**Figure 3:**
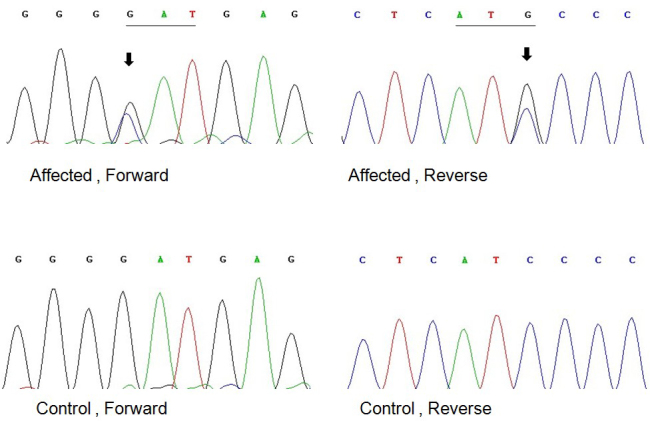
Forward and reverse sequence analysis of the affected and unaffected individuals in a Chinese family with autosomal dominant congenital cataract (ADCC), showing a G139C mutation of the connexin 50 gene (black arrows).

Through bidirectional sequencing of the coding regions of the candidate genes, we identified a heterozygous change, G>C, at position 139(c. 139G>C) of the connexin 50 (*GJA8*) gene, leading to the replacement of a wild-type aspartic acid with a histidine at the 47th amino acid position (p. D47H; Figure 3). No amino acid substitution or mutation was found in the other candidate genes. Using the sequencing method, this D47H substitution cosegregated with all six affected individuals, whereas it was not present in the unaffected family members or in 100 unrelated Chinese controls without cataracts.

### Bioinformatics analysis

Computational protein analysis of D47H Cx50 revealed the following results: PolyPhen analysis produced a score of 1.000, which is predicted to be “probably damaging.” Align-GVGD showed a score of GV0.00, GD81.24, which belongs to class C65 and means “most likely to interfere with function.” Finally, the SIFT method revealed a score of 0.02, meaning the substitution is intolerant. As the isolated predictive value of these programs can be increased by their combination [19], these results indicated the D47H substitution was likely deleterious and possibly contributed to the disease.

## Discussion

In this study, we identified a novel mutation within the connexin 50 gene (*GJA8*) in a four-generation Chinese pedigree with autosomal dominant cataract. The lens is an avascular organ that transmits and focuses light images on the retina. The lens has developed an extensive cell–cell communication system using connexins to maintain transparency [20]. Intercellular gap junction communication, which is essential for this system and integral to the internal microcirculation of the lens, provides pathways for metabolites, ions and signaling molecules, and other molecules smaller than 1 kDa [21, 22].

Connexin proteins are membrane proteins containing four transmembrane domains (M1, M2, M3, and M4), two extracellular loops (E1 and E2), and three intracellular regions (the NH_2_-terminus, a cytoplasmic loop, and the COOH-terminus) [23]. Six connexin protein subunits oligomerize to form one hemichannel (also called connexon) that then are transported to the plasma membrane. The hemichannel consists of a positively charged cytoplasmic entrance, a pore funnel, a negatively charged transmembrane pathway, and an extracellular cavity [24]. A gap junction channel is formed by the docking of the extracellular loops of two hemichannnels from two adjacent cells [25]. Intercellular gap junction channels are formed by at least three different connexin protein subunits in the human lens, connexin 43 (*GJA1*) [26], connexin 46 (*GJA3*) [27], and *GJA8* [28]. Cx43 is restrictively expressed in the lens epithelial cells, Cx46 is primarily expressed in lens fiber cells, and Cx50 is expressed in lens epithelial and fiber cells [29, 30]. Diverse gap junction channels formed by Cx46 and Cx50 subunits are important for the differentiation, elongation, and maturation of lens fiber cells [31]. Recent genetic studies showed that Cx46 is important for lens transparency while Cx50 is essential for lens growth and transparency [32].

The coding region of *GJA8* is contained within one exon, which encodes a polypeptide containing 432 amino acids. To date, more than 24 mutations in the connexin 50 gene in humans and mice have been reported to induce genetic cataracts, which were summarized by Wang et al. recently [33].

**Figure 4:**
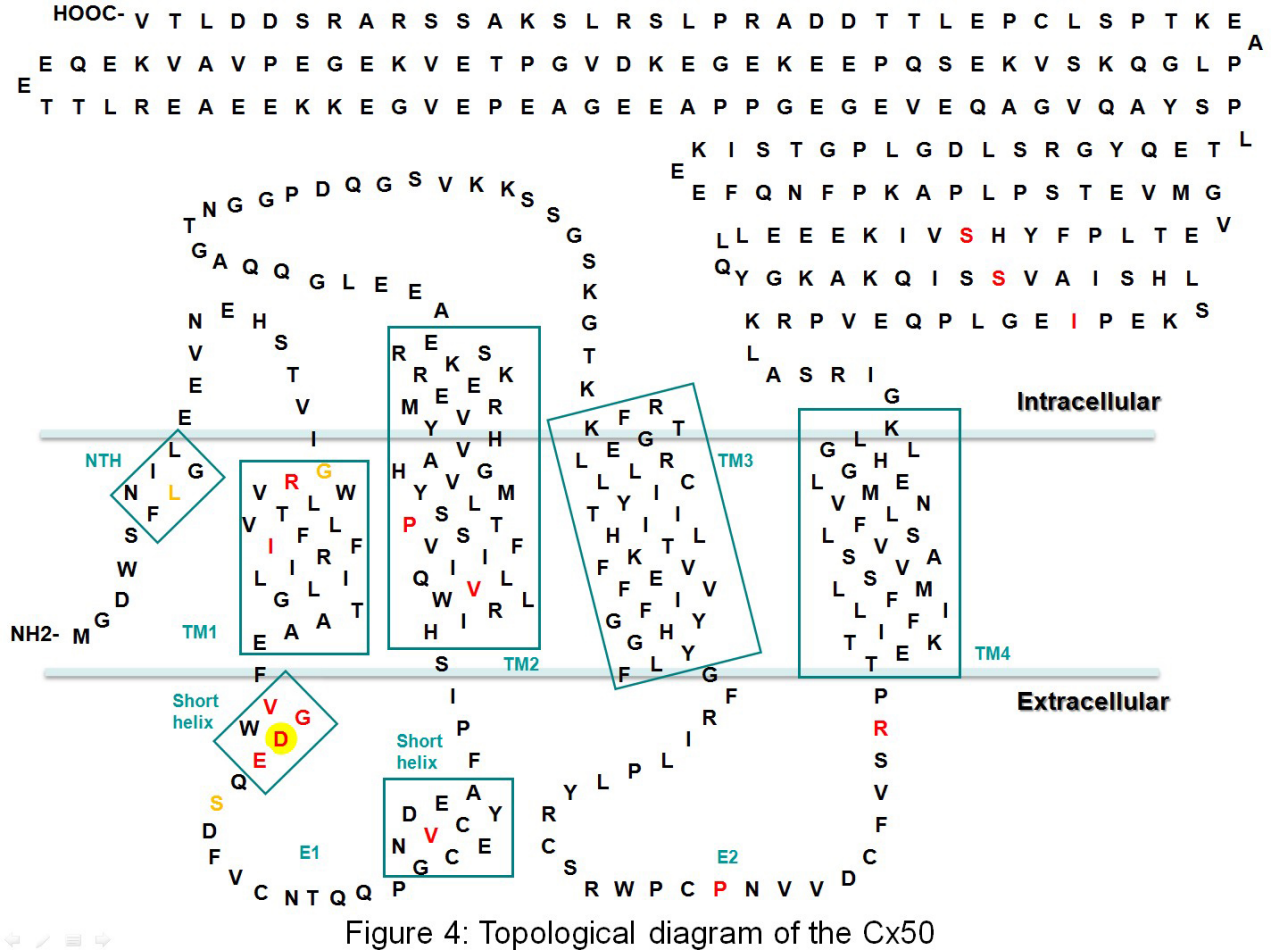
Boxes indicate regions with a helical secondary structure denoted as NTH, TM1, TM2, TM3, TM4, and a short helix in E1. The membrane region is depicted in light blue. Red letters: Previous connexin 50 gene mutations with congenital cataract in humans: R23T, I31T, V44E, W45S, G46V, D47N, D47Y, E48K, V64G, V79L, P88Q, P88S, P189L, R198W, R198Q, I247M, S258F, and S276F. Yellow letters: Previous connexin 50 gene mutations in the mouse: L7Q, G22R (Lop10 mouse), D47A (nuclear opacity 2, No 2 mouse), and S50P (L1 mouse). Red letter in yellow circle: the new mutation D47H in this family.

The observed 139G>C substitution replaces the negatively charged aspartic acid with positively charged histidine at position 47 in association with the congenital cataract in the present study. Maeda’s structural studies of the connexin gap junction channel indicated that Asp 47 is located in the beginning of extracellular loop E1 of Cx50 (Figure 4). In addition, pore structure analysis of the Cx26 gap junction channel showed that Asp 46 and Asp 50, highly conserved residues in the connexin family, face the pore interior and create a negatively charged transmembrane path approximately at the height of the extracellular membrane surface. Along with the pore funnel, these two regions possibly contribute to the charge selectivity and probably to the size restriction, considering the charge character and the pore diameter [24]. Until now, several amino acid substitutions at position 47 or nearby amino acids have been identified in the connexin 50 gene in association with congenital cataracts in mice and humans, including Cx50V44E [34], Cx50W45S [35], Cx50G46V [36], Cx50D47Y [37], Cx50D47N [33], Cx50E48K [38], mCX50D47A [39], and mCX50S50P [40].

Except for Cx50G46V, which enhances the hemichannel function and leads to cell death [36], most CX50 mutants exhibit impaired trafficking and/or lack of function. Research on Cx50E48K revealed that this mutation affects gap junctions but not the hemichannel function of Cx50, in a dominant negative manner when paired with wild-type Cx50, but has no such effect on Cx46 [41].

Study of the nuclear opacity 2 mouse shows that mutation D47A fails to form functional junctions and is unable to inhibit wild-type Cx46 or Cx50 junctional conductance when expressed in *Xenopus* oocyte pairs [39]. In the same way, the D47N [42] mutation in humans acted as a loss-of channel-function mutation without dominant negative inhibition of wild-type Cx50. Combining the Align-GVGD, PolyPhen, and SIFT results, we believe that the D47H mutation might also be explained by the loss-of-function mechanism, as the mutations D47A and D47N were. Further experiments on this cataract-related genetic defect will improve our understanding of the mechanism of cataract formation and illuminate the role of the connexin family in the lens.

## References

[1] Apple DJ, Ram J, Foster A, Peng Q (2000). Elimination of cataract blindness: A global perspective entering the new millenium.. Surv Ophthalmol.

[2] Rahi JS, Sripathi S, Gilbert CE, Foster A (1995). Childhood blindness in india: Causes in 1318 blind school students in nine states.. Eye (Lond).

[3] Gilbert C, Foster A (2001). Childhood blindness in the context of vision 2020--the right to sight.. Bull World Health Organ.

[4] Francis PJ, Berry V, Bhattacharya SS, Moore AT (2000). The genetics of childhood cataract.. J Med Genet.

[5] Vogt G, Puho E, Czeizel AE (2005). Population-based case-control study of isolated congenital cataract.. Birth Defects Res A Clin Mol Teratol.

[6] Hejtmancik JF (2008). Congenital cataracts and their molecular genetics.. Semin Cell Dev Biol.

[7] Vanita, Singh D (1999). Genetic and segregation analysis of congenital cataract in the indian population.. Clin Genet.

[8] Shiels A, Bennett TM, Hejtmancik JF (2010). Cat-map: Putting cataract on the map.. Mol Vis.

[9] Reddy MA, Francis PJ, Berry V, Bhattacharya SS, Moore AT (2004). Molecular genetic basis of inherited cataract and associated phenotypes.. Surv Ophthalmol.

[10] Devi RR, Yao W, Vijayalakshmi P, Sergeev YV, Sundaresan P, Hejtmancik JF (2008). Crystallin gene mutations in indian families with inherited pediatric cataract.. Mol Vis.

[11] Yao K, Jin C, Zhu N, Wang W, Wu R, Jiang J, Shentu X (2008). A nonsense mutation in crygc associated with autosomal dominant congenital nuclear cataract in a chinese family.. Mol Vis.

[12] Yao K, Li J, Jin C, Wang W, Zhu Y, Shentu X, Wang Q (2011). Characterization of a novel mutation in the crybb2 gene associated with autosomal dominant congenital posterior subcapsular cataract in a chinese family.. Mol Vis.

[13] Yao K, Wang W, Zhu Y, Jin C, Shentu X, Jiang J, Zhang Y, Ni S (2011). A novel gja3 mutation associated with congenital nuclear pulverulent and posterior polar cataract in a chinese family.. Hum Mutat.

[14] Zhu Y, Shentu X, Wang W, Li J, Jin C, Yao K (2010). A chinese family with progressive childhood cataracts and ivs3+1g>a cryba3/a1 mutations.. Mol Vis.

[15] Yu Y, Li J, Xu J, Wang Q, Yao K (2012). Congenital polymorphic cataract associated with a g to a splice site mutation in the human beta-crystallin gene crybetaa3/a1.. Mol Vis.

[16] Santana A, Waiswol M, Arcieri ES, Cabral de Vasconcellos JP, Barbosa de Melo M (2009). Mutation analysis of cryaa, crygc, and crygd associated with autosomal dominant congenital cataract in brazilian families.. Mol Vis.

[17] Mathe E, Olivier M, Kato S, Ishioka C, Hainaut P, Tavtigian SV (2006). Computational approaches for predicting the biological effect of p53 missense mutations: A comparison of three sequence analysis based methods.. Nucleic Acids Res.

[18] Ng PC, Henikoff S (2003). Sift: Predicting amino acid changes that affect protein function.. Nucleic Acids Res.

[19] Chan PA, Duraisamy S, Miller PJ, Newell JA, McBride C, Bond JP, Raevaara T, Ollila S, Nystrom M, Grimm AJ, Christodoulou J, Oetting WS, Greenblatt MS (2007). Interpreting missense variants: Comparing computational methods in human disease genes cdkn2a, mlh1, msh2, mecp2, and tyrosinase (tyr).. Hum Mutat.

[20] Goodenough DA (1979). Lens gap junctions: A structural hypothesis for nonregulated low-resistance intercellular pathways.. Invest Ophthalmol Vis Sci.

[21] Saez JC, Berthoud VM, Branes MC, Martinez AD, Beyer EC (2003). Plasma membrane channels formed by connexins: Their regulation and functions.. Physiol Rev.

[22] Mathias RT, Rae JL, Baldo GJ (1997). Physiological properties of the normal lens.. Physiol Rev.

[23] Yeager M, Harris AL (2007). Gap junction channel structure in the early 21st century: Facts and fantasies.. Curr Opin Cell Biol.

[24] Maeda S, Nakagawa S, Suga M, Yamashita E, Oshima A, Fujiyoshi Y, Tsukihara T (2009). Structure of the connexin 26 gap junction channel at 3.5 a resolution.. Nature.

[25] Mathias RT, White TW, Gong X (2010). Lens gap junctions in growth, differentiation, and homeostasis.. Physiol Rev.

[26] Beyer EC, Paul DL, Goodenough DA (1987). Connexin43: A protein from rat heart homologous to a gap junction protein from liver.. J Cell Biol.

[27] Paul DL, Ebihara L, Takemoto LJ, Swenson KI, Goodenough DA (1991). Connexin46, a novel lens gap junction protein, induces voltage-gated currents in nonjunctional plasma membrane of xenopus oocytes.. J Cell Biol.

[28] White TW, Bruzzone R, Goodenough DA, Paul DL (1992). Mouse cx50, a functional member of the connexin family of gap junction proteins, is the lens fiber protein mp70.. Mol Biol Cell.

[29] Gong X, Li E, Klier G, Huang Q, Wu Y, Lei H, Kumar NM, Horwitz J, Gilula NB (1997). Disruption of alpha3 connexin gene leads to proteolysis and cataractogenesis in mice.. Cell.

[30] Rong P, Wang X, Niesman I, Wu Y, Benedetti LE, Dunia I, Levy E, Gong X (2002). Disruption of gja8 (alpha8 connexin) in mice leads to microphthalmia associated with retardation of lens growth and lens fiber maturation.. Development.

[31] Gong X, Cheng C, Xia CH (2007). Connexins in lens development and cataractogenesis.. J Membr Biol.

[32] Sellitto C, Li L, White TW (2004). Connexin50 is essential for normal postnatal lens cell proliferation.. Invest Ophthalmol Vis Sci.

[33] Wang L, Luo Y, Wen W, Zhang S, Lu Y (2011). Another evidence for a d47n mutation in gja8 associated with autosomal dominant congenital cataract.. Mol Vis.

[34] Devi RR, Vijayalakshmi P (2006). Novel mutations in gja8 associated with autosomal dominant congenital cataract and microcornea.. Mol Vis.

[35] Vanita V, Singh JR, Singh D, Varon R, Sperling K (2008). A novel mutation in gja8 associated with jellyfish-like cataract in a family of indian origin.. Mol Vis.

[36] Minogue PJ, Tong JJ, Arora A, Russell-Eggitt I, Hunt DM, Moore AT, Ebihara L, Beyer EC, Berthoud VM (2009). A mutant connexin50 with enhanced hemichannel function leads to cell death.. Invest Ophthalmol Vis Sci.

[37] Lin Y, Liu N-n, Lei C-t, Fan Y-c, Liu X-q, Yang Y, Wang J-f, Liu B, Yang Z-l (2008). A novel gja8 mutation in a chinese family with autosomal dominant congenital cataract.. Zhonghua Yixue Yichuanxue Zazhi.

[38] Berry V, Mackay D, Khaliq S, Francis PJ, Hameed A, Anwar K, Mehdi SQ, Newbold RJ, Ionides A, Shiels A, Moore T, Bhattacharya SS (1999). Connexin 50 mutation in a family with congenital "zonular nuclear" pulverulent cataract of pakistani origin.. Hum Genet.

[39] Xu X, Ebihara L (1999). Characterization of a mouse cx50 mutation associated with the no2 mouse cataract.. Invest Ophthalmol Vis Sci.

[40] Xia CH, Liu H, Cheung D, Cheng C, Wang E, Du X, Beutler B, Lo WK, Gong X (2006). Diverse gap junctions modulate distinct mechanisms for fiber cell formation during lens development and cataractogenesis.. Development.

[41] Banks EA, Toloue MM, Shi Q, Zhou ZJ, Liu J, Nicholson BJ, Jiang JX (2009). Connexin mutation that causes dominant congenital cataracts inhibits gap junctions, but not hemichannels, in a dominant negative manner.. J Cell Sci.

[42] Arora A, Minogue PJ, Liu X, Addison PK, Russel-Eggitt I, Webster AR, Hunt DM, Ebihara L, Beyer EC, Berthoud VM, Moore AT (2008). A novel connexin50 mutation associated with congenital nuclear pulverulent cataracts.. J Med Genet.

